# How Do Korean Secondary School Teachers Perceive Psychological Burnout in Their Teaching Careers?

**DOI:** 10.3390/bs14121210

**Published:** 2024-12-17

**Authors:** Taeeun Shim, Mikyung Jun, Song Yi Lee

**Affiliations:** 1 Department of Education, College of Education, Dongguk University, Seoul 04620, Republic of Korea; shim2593@dongguk.edu; 2 Department of Home Economics Education, College of Education, Dongguk University, Seoul 04620, Republic of Korea; 3 Department of Counselling and Coaching, Dongguk University, Seoul 04620, Republic of Korea

**Keywords:** Korean secondary school teachers, teaching career, burnout, Q methodology, perception

## Abstract

The psychological burnout experienced by teachers is not merely a personal issue; it is a significant social problem that impacts the entire educational environment. This study utilised Q methodology to identify the subjective perceptions of psychological burnout among Korean secondary school teachers and then analysed the characteristics of these perception types. An analysis of 34 statements and a P sample of 30 teachers resulted in four types of perceptions regarding teachers’ psychological burnout: ‘burnout due to damaged self-esteem’ (Type 1), ‘burnout due to organisational neglect’ (Type 2), ‘burnout due to excessive role demands’ (Type 3) and ‘burnout due to disrespectful behaviour’ (Type 4). This study’s meaningfulness is in its classification of teachers’ psychological burnout into types and its exploration of the types’ features. The results can also help in developing specific intervention programmes for each type.

## 1. Introduction

Recently, burnout has rapidly increased due to changes in lifestyles and human relationships and the rising complexity of organisational societies worldwide. This trend negatively affects individuals’ well-being and job performance [1,2]. Among various professions, teaching is one of the most affected by burnout. Teachers face emotional exhaustion caused by prolonged working hours, heavy workloads, and challenging student behaviour [3,4]. Burnout, characterised by physical and mental fatigue, lethargy, self-loathing and even depression, often results from using excessive energy during job tasks [5,6]. In particular, teachers often experience burnout due to student misconduct and conflicts with parents, regardless of school level [7,8].

In addition to these factors, changes in school organisation, culture and the digital environment create challenges in curriculum development and student guidance. These challenges further contribute to teacher burnout, decreasing job satisfaction and increasing job turnover rates [5,9,10]. Burnout can reduce passion for teaching and job satisfaction, ultimately lowering the quality of education and student engagement. Burned-out teachers may leave the profession, worsening teacher shortages and deepening educational inequality [10,11]. In addition, teacher burnout has broader implications. It hinders the teacher’s ability to deliver effective instruction and negatively impacts student academic performance and emotional well-being. There is also an effect on the overall learning environment and teacher–student interactions, creating a cycle of dissatisfaction for all involved [12].

In South Korea, high academic achievement expectations and intense competition for university entrance are significant stressors for teachers. Factors such as high parental expectations, frequent changes in educational policies and rapid digital transformation intensify Korean teachers’ burnout experiences [13]. Given these challenges, it is crucial to explore teacher burnout through their subjective experiences. Thus, this study employs Q methodology, a systematic approach for investigating subjective perspectives, to categorise teachers’ personal experiences of burnout into types. By uncovering the diversity of perceptions, it provides valuable insights into the complex nature of teacher burnout. Scholars can use the findings to develop targeted prevention and management strategies tailored to each type of burnout.

## 2. Literature Review

### 2.1. Concepts and Components of Psychological Burnout

People commonly understand burnout as a combination of emotional exhaustion, depersonalisation, and a reduced sense of personal accomplishment. While the Maslach Burnout Inventory (MBI), introduced by Maslach and Jackson (1981), laid the foundation for burnout measurement, recent studies have refined this framework to address diverse occupational settings, including education [14,15].

Emotional exhaustion, marked by persistent fatigue and depleted energy, arises when individuals face sustained stressors without sufficient recovery. For teachers, this often results from societal pressures and role overload, such as handling excessive administrative responsibilities and managing students’ character development [16,17,18,19]. Depersonalisation, characterised by a detached and cynical attitude, frequently occurs when teachers encounter disrespect or inadequate support from students, parents or administrators [9,20]. The increasing emphasis on student rights and a customer-oriented approach to education intensifies these challenges and contributes to burnout [21,22]. In addition, there is a close link between reduced sense of personal accomplishment and self-efficacy. Teachers with lower self-efficacy are less likely to meet job expectations, which heightens burnout risk. Conversely, higher self-efficacy supports resilience and enhances educational outcomes [23,24].

In South Korea, unique cultural and organisational dynamics further shape burnout experiences. Teachers must balance evolving societal expectations, including heightened parental demands and the rapid integration of digital technologies [25,26,27,28]. Initiatives like parental education programs and efforts to redistribute workloads have shown promise in alleviating these pressures [14].

### 2.2. Shifting Teaching Profession and Challenges Faced by Teachers

Teaching in Korea was once regarded as a highly respected and attractive profession. Kang and Hong (2008) [29] attributed the achievements of Korean students to adequate class hours and preparation time and a structure that recognised teachers as professionals. However, social changes and shifts in the educational environment have altered this perception. Thus, people no longer see teaching as a desirable job.

According to the 2018 Early Childhood Development Teaching and Learning International Survey (ECD TALIS), Korean secondary school teachers reported low job satisfaction and self-efficacy compared to the Organisation for Economic Co-operation and Development (OECD) average. Korea ranked third in the proportion of teachers expressing regret about choosing teaching as a profession, following Saudi Arabia and Portugal (OECD, 2018) [30]. Korean teachers identified excessive administrative work, maintaining classroom order and handling parental complaints as primary stressors. Additionally, they were more likely to report threats or verbal abuse from students and parental conflicts as high stress factors than teachers from other countries [31]. To address these challenges, South Korea implemented measures in September 2023 to support teachers’ mental health. These included expanded access to psychological assessments and counselling services [32].

The challenges faced by teachers are not unique to Korea. In the United Kingdom, the Teacher Well-being Report revealed that 77% of teachers experience mental health issues, with 46% reporting insomnia or other sleep disorders caused by work-related stress. Major causes of burnout include excessive workloads, poor work–life balance and a diminished sense of professional value (Education Support, 2024) [33]. Teachers in the United States report high levels of occupational stress driven by unrealistic expectations, overwhelming responsibilities and insufficient support. The COVID-19 pandemic exacerbated these issues by adding new responsibilities related to remote learning and virtual student engagement [34,35]. In addition, Australia identifies key stressors as workforce shortages, insufficient leadership support and time constraints. These factors contribute to high turnover rates and negatively impact the well-being of children and adolescents [36].

A comparative analysis of teacher burnout in South Korea, the United Kingdom, the United States and Australia highlights similarities and differences. Teachers universally report high stress levels that negatively affect educational quality and retention rates. However, the specific sources of burnout differ by country. In Korea, stress arises from handling parental complaints and verbal abuse from students. In the United Kingdom, a poor work–life balance and diminished professional value are primary concerns, while in the United States, teachers struggle with excessive responsibilities and adapting to remote learning. Finally, in Australia, resource shortages and leadership challenges are key issues. These findings underscore the need for tailored solutions that address the cultural and contextual specificities of each country.

Therefore, this study explores the types of subjective perceptions of burnout among Korean teachers and asks the following research questions:

1. How do Korean secondary school teachers perceive psychological burnout?
2. What are the characteristics of the types of psychological burnout as perceived by Korean secondary school teachers?

## 3. Methods

This study applies the Q methodology to explore the perceptions of psychological burnout among Korean secondary school teachers. Q methodology is a mixed research approach that focuses on measuring the diversity of human subjectivity, including mindsets, preferences, opinions and attitudes [37,38]. This framework can present distinct perspectives on a given topic, provide an overview of historical and theoretical foundations and describe underlying perceptions [39,40]. This method emphasises phenomenological generalisation over statistical generalisation, enabling the categorisation of subjective viewpoints within a population to present diverse perspectives on the subject [41].

In Q methodology, researchers select statements related to a specific topic, and participants rank these statements based on their level of agreement. The researchers then conduct factor analysis on these data to group similar viewpoints among respondents and derive representative perspectives for each group. The process focuses on identifying patterns in subjective data, making it effective for uncovering significant psychological experiences [42]. Therefore, Q methodology is well-suited for understanding teachers’ subjective perceptions of burnout.

**Figure 1:**
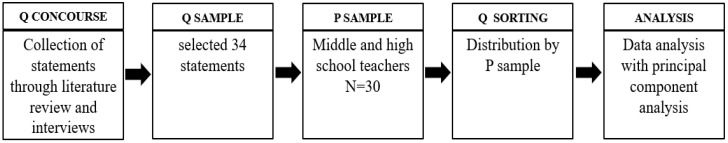
Research process.

This study followed the Q methodology steps shown in Figure 1.

### 3.1. Q Concourse

The Q concourse is a collection of statements encompassing all perspectives, including research participants’ subjective thoughts and opinions regarding the topic under study [43,44,45]. This study developed the Q concourse by following a structured process, which included two main steps: (1) extracting statements from the academic literature and (2) gathering additional statements through written interviews.

First, we reviewed the literature to identify statements related to psychological burnout. The keywords used included ‘measurement tools for psychological burnout’, ‘emotional exhaustion of teachers’, ‘job satisfaction’, ‘job stress’ and ‘attitudes towards students that induce burnout’ [21,46,47,48,49]. Based on these criteria, we initially selected 121 statements representing various aspects of teachers’ psychological burnout.

Second, we conducted written interviews with middle and high school teachers to gather additional statements and perspectives. We recruited participants via email, informed them about the study and invited them to participate after they gave informed consent. To capture diverse experiences, we used purposive sampling to select five middle school teachers and five high school teachers. The participants included five males and five females, with five teachers from Seoul and five from Gyeonggi Province. Their teaching subjects included English (3), home economics (2), social studies (1), mathematics (1), Korean language (1), science (1) and ethics (1).

The written interviews included questions such as the following:

- What types of psychological burnout are teachers currently experiencing?
- If you have experienced burnout, can you provide specific examples?
- How did you overcome psychological burnout?

We repeatedly read and analysed the responses to ensure that participants’ statements reflected their subjective experiences. As a result, we derived 220 statements from the interviews and combined these with the 121 statements from the literature review, resulting in a total of 341 statements for the Q concourse.

### 3.2. Q Sample

The Q sample refers to the subset of statements extracted from the Q concourse [50,51]. To create the Q sample, we followed a three-step process: categorisation, review and final selection.

First, the research team categorised the 341 statements from the Q concourse into thematic groups, such as causes of burnout, prevention methods, policies, job stress, attitudes towards students, administrative support, frustration, relationship dissatisfaction and lack of personal achievement. After categorising the statements, we selected those that comprehensively and diversely represented opinions within each categorised domain. Each researcher independently chose statements, and we included those selected by two or more researchers. This process narrowed the statements down to 99.

Second, four middle and high school teachers reviewed the 99 statements to assess their relevance and appropriateness for the study. We excluded statements judged as irrelevant or redundant by more than three participants, resulting in the elimination of 32 statements.

Finally, the researchers further reviewed the remaining 67 statements to confirm their relevance, representativeness and comprehensiveness and selected statements agreed upon by all researchers. This final step reduced the list to 34 statements, which constituted the Q sample for the study.

### 3.3. P Sample

The P-sample refers to the respondents participating in the Q-sorting process. In Q methodology, sampling aims to identify diverse perspectives related to the research topic rather than achieving statistical generalisation. Researchers typically use purposive sampling to select participants who can provide meaningful insights, with a sample size of 30 to 60 participants being sufficient for this purpose [43,52].

To ensure representativeness within the cultural context, we purposively selected participants in this study based on key demographic and professional criteria, including school level, gender, school type, teaching years, geographic region, teaching subject and age. These criteria reflect the diversity of teachers’ experiences and backgrounds relevant to the research topic. For example, participants included 17 middle school teachers and 13 high school teachers to capture differences between school levels and 14 teachers from Seoul and 16 from Gyeonggi Province to account for geographic variation. Similarly, the inclusion of 17 public school teachers and 13 private school teachers ensured representation across different school systems. Further, we considered teaching experience, with 18 participants having more than 10 years of experience and 12 having less than 10 years. These demographic details, summarised in Table 1, provide a robust foundation for understanding diverse perspectives on teacher burnout in the Korean context.

### 3.4. Q Sorting

**Figure 2:**
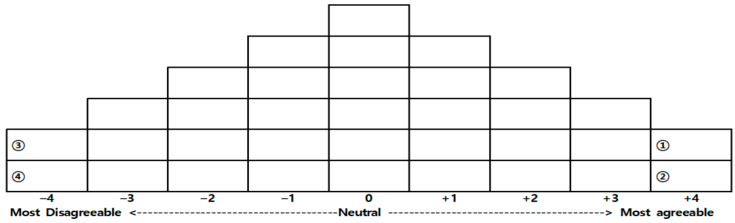
Sample grid for Q sorting pattern.

In Q sorting, participants sort the Q sample statements based on their degree of agreement. In this study, participants arranged 34 statement cards in a forced distribution format, ranging from strongly disagree (−4) to strongly agree (+4), as illustrated in Figure 2 [53].

The Q sorting process involved three main steps.

First, participants read the 34 statements multiple times to become familiar with all of them. Second, they sorted the statement cards into three categories based on whether they agreed or disagreed with each statement: agree, disagree and neutral. Third, they distributed the cards sequentially on the Q sorting sheet, placing those they most agreed with on the right end (+4) and those they most disagreed with on the left end (−4).

Additionally, we asked participants to explain their choices for the two statements that they agreed with most (positions +4, ① and ②) and the two statements that they disagreed with most (positions −4, ③ and ④). This qualitative feedback provided deeper insights into their perspectives and enhanced the richness of the data. Each sorting session took approximately 40–50 min to complete.

### 3.5. Data Analysis

We analysed the collected Q sorting data using KADE v1.2.1, a desktop application designed for Q methodology analysis. This software supports multiple operating systems, including Microsoft Windows, Apple macOS and Linux [54].

We applied principal component analysis to group similar responses into factors and used the varimax rotation method to improve interpretability. Using a scree plot and cumulative variance, we identified four factors for this study; we calculated Z-scores and Q-sort values for each type and statement.

Moreover, to interpret the characteristics of each factor, we reviewed participants’ qualitative explanations for the statements with which they agreed and disagreed most. This approach provided a comprehensive understanding of each type’s unique perspectives and their underlying rationale.

## 4. Results

### 4.1. Preliminary Results

The analysis resulted in four perception factors of burnout experienced by Korean secondary school teachers. Each factor explains the characteristics of the grouped individuals by clustering the P sample based on similar opinions, thoughts and attitudes.

The cumulative variance of these four factors was 54%, with a value above 50% considered to have high explanatory power [45]. An examination of the explanatory power by type revealed that Factors 1, 2, 3 and 4 accounted for 25%, 13%, 9% and 7%, respectively. Lastly, the eigenvalues for each factor were 7.3738, 3.7675, 2.6625 and 2.0241, respectively (Table 2).

### 4.2. Characteristics of Psychological Burnout in Secondary School Teachers

As a result of the factor analysis [55], we classified the four factors representing the characteristics of teacher burnout types (Table 3).

#### 4.2.1. Type 1: Burnout Due to Damaged Self-Esteem

Type 1 teachers expressed shame and frustration due to the rude attitudes of parents and students, being treated as service providers by the school and their perceived lack of influence over students and parents. These factors contributed significantly to their burnout. Thus, we named the burnout of Type 1 teachers ‘burnout due to damaged self-esteem’.

Type 1 teachers agreed with statements such as ‘I feel frustrated when student misconduct is blamed on teachers’ (S7), ‘I feel a sense of shame when parents or students view the school as a service institution’ (S34), ‘I feel humiliated by the rude attitudes of students or parents’ (S3) and ‘I feel frustrated when I think I lack competence as a teacher’ (S32). However, they disagreed with statements like ‘I am exhausted by the feeling that people see me only as someone with teaching duties’ (S4), ‘I get angry when fellow teachers disregard my subject’ (S9) and ‘The excessive emphasis on student rights compared to teacher authority is problematic’ (S6).

P11 and P14, with high factor weights, stated, ‘The essence of the problems caused by students lies with the students and their families, and it is wrong to ignore this and place full responsibility on the teachers’. They also mentioned, ‘Guiding and managing students is mentally stressful, and linking student problems to the teacher’s qualifications increases frustration’. P4 noted, ‘Parents interpret teachers’ criticisms of student behaviour as the teacher’s fault and protest, which psychologically intimidates teachers who have to persuade such parents’. Lastly, P18, a first-year teacher, responded, ‘I experience burnout when arrogant parents treat me as an incompetent teacher lacking ability and qualifications and even try to teach me’.

An examination of the Q-sort values revealed that Type 1 teachers agreed strongly with S34 (+4) and S32 (+3), unlike other types. Participants who chose these statements said the following:

*My self-esteem drops when asked to take care of trivial matters for each student. Additionally, I feel ashamed when they act like consumers receiving care services. I wonder if they make such requests because of my lack of competence, which leads to burnout* (P11, P14).

Type 1 participants disagreed more strongly with S4 (−4) compared to other types, feeling confident that others did not merely see them as duty performers (P27). For S6 (−3), while other types agreed, Type 1 teachers did not, stating, ‘The causes of infringement on teacher authority should not solely emphasise student rights; other issues should also be considered’ (P11, P14).

For Type 1 teachers, psychological burnout stems from feelings of humiliation due to damage to their self-esteem as teachers. The rude attitudes of students and parents cause deep frustration. They experience psychological loss and identity confusion when others regard teaching merely as a service provision. In other words, they feel shame and humiliation when students and parents do not respect their influence as professionals, and they experience psychological intimidation when others undervalue their qualifications as teachers. Modern societal changes increasingly challenge the cultural emphasis on teacher authority and respect in South Korea, amplifying these experiences.

#### 4.2.2. Type 2: Burnout Due to Organisational Neglect

Type 2 teachers expressed a deep sense of helplessness, citing burnout as a result of the teaching organisation or administrators failing to protect them due to indifference. We classified the burnout of these teachers as ‘burnout due to organisational neglect’.

Type 2 teachers agreed with statements such as ‘The administrator’s passive response to complaints lowers motivation’ (S12), ‘There’s not much I can do for fellow teachers who suffer from infringement of their authority in the school field’ (S11), ‘I feel frustrated with a teaching organisation that does not prioritise the well-being of teachers’ (S1) and ‘It is unfair for administrators to assign tasks unilaterally without considering my aptitude or circumstances’ (S29). However, they disagreed with statements like ‘Distrust in public education causes psychological burnout for teachers’ (S30), ‘I feel anxious about how long I can continue my teaching career’ (S14) and ‘To prevent teacher burnout, I cannot correct every mistake students make’ (S15).

P26, with the highest factor weight, stated, ‘I experience psychological burnout when students are disrespectful and behave rudely towards teachers and when parents make unreasonable demands to write student records in their desired way’. P2 mentioned, ‘If teachers do not act as teachers because they are wary of students or parents, this place is no different from a cram school that only delivers knowledge’. In addition, P3 states:

When facing difficulties, the lack of support and assistance from administrators or the school further intimidates teachers. Therefore, higher institutions like the education office can prevent teachers from leaving the profession due to helplessness by maintaining a basic attitude of respect towards teachers.

An examination of the Q-sort values revealed that Type 2 teachers agreed more strongly with S11 (+4) and S1 (+3) compared to other types. Participants said, ‘Teachers are a special group, so the social standards for teachers are strict. Therefore, teachers must rely on and trust each other, but it is most frustrating when the teacher community or other teachers trample on their well-being’ (P20). Type 2 teachers showed relatively high agreement with S9 (+2), but other types showed disagreement. This finding indicates that teacher burnout occurs when fellow teachers infringe upon their authority. Unlike other types, Type 2 strongly agreed with S29 (+3), indicating that administrators’ unilateral actions are a factor in teacher burnout, a distinctive perception characteristic of this type.

Type 2 experiences burnout due to institutional issues, particularly when the school organisation shows no interest in teachers’ problems and lacks appropriate responses. This situation reflects the cultural context in South Korea, where hierarchical structures and administrative indifference can exacerbate feelings of helplessness among teachers.

#### 4.2.3. Type 3: Burnout Due to Excessive Role Demands

Type 3 teachers emphasise burnout caused by unrealistic role expectations, poor treatment and the irrationality of holding teachers responsible for students’ character development. Thus, we named Type 3 ‘burnout due to excessive role demands’.

Type 3 teachers agreed with statements such as ‘If teachers’ salaries and treatment improve, teacher authority will increase’ (S31), ‘I feel frustrated when student misconduct is blamed on teachers’ (S7), ‘I feel humiliated by the rude attitudes of students or parents’ (S3) and ‘It’s wrong to make teachers responsible for students’ character development’ (S16). However, they disagreed with statements like ‘I feel frustrated when I think I lack competence as a teacher’ (S32) and ‘Unreasonable demands to include specific content in student records diminish my motivation’ (S23).

P28, with a high factor weight, stated, ‘When guiding students in their daily lives, it is often difficult to respond rationally. I also experience psychological burnout from repeatedly encountering situations where a simple apology follows rude behaviour’. P25 mentioned, ‘Parents say student problems are due to friends, the school, or teachers, but they seem unaware that this attitude hinders students’ growth. The reality of parents focusing only on students getting into good universities leads to psychological burnout’.

An examination of the Q-sort values revealed that Type 3 teachers agreed with S31 (+4) and S5 (+1), unlike the other three types. Participants said, ‘Improving teachers’ treatment is vital for enhancing teacher authority, job satisfaction and social status. This improvement will also attract talented individuals to the teaching profession, thereby increasing teachers’ professionalism’ (P27). Type 3 strongly disagreed with S32 (−4) and S23 (−3), unlike other types, indicating that Type 3 teachers are confident in their professionalism but suffer burnout when experiencing unreasonable responsibilities.

Type 3 teachers experience burnout due to social expectations and role overload, feeling burnout from the unreasonable demand that they are responsible for students’ character development beyond education. Rude attitudes from students and parents particularly exacerbate emotional burnout. In South Korea’s cultural context, societal expectations heavily burden teachers by demanding they fulfil multiple roles beyond academic instruction.

#### 4.2.4. Type 4: Burnout Due to Disrespectful Behaviour

Type 4 teachers emphasise burnout caused by student indifference, the excessive emphasis on student rights and disrespectful behaviours from students in the classroom. We termed this type ‘burnout due to disrespectful behaviour’.

Type 4 teachers agreed with statements such as ‘The excessive emphasis on student rights compared to teacher authority is problematic’ (S6), ‘I experience psychological burnout when I see students openly sleeping during class’ (S27) and ‘I experience psychological burnout when I see students doing academy homework during class’ (S26). However, they disagreed with statements like ‘I experience psychological burnout when I lose confidence in my work’ (S13), ‘I feel burned out when others demand only exemplary behaviour from teachers’ (S18)’ and ‘Schools should not offer teachers’ personal contact information to students or parents (S19).

P8, with the highest factor weight, stated the following:

I work after hours and on weekends to provide better lessons, but it becomes difficult when students make dismissive comments like “My academy teacher said this”. Additionally, I experience burnout when the demands of students and parents increase while trust in teachers decreases.

In addition, P5 commented:

Providing high-quality lessons is the role of a teacher. I engage in endless research and reflection to improve the quality of education. However, I feel helpless when I encounter students who give up on participating in class and ignore the teacher, such as sleeping during lessons.

An examination of the Q-sort values revealed that Types 2 and 3 disagreed with S27 (+4), while Type 1 indicated that burnout occurs due to lowered self-esteem. P23, a Type 4, stated, ‘When I see students sleeping in class after attending academy lessons, or when students who have lived abroad ignore English lessons, or when I am unable to discipline students, I question whether I am truly a teacher’. Type 4 strongly agreed with S6 (+4), while Type 1 did not agree. Additionally, unlike other types, Type 4 did not agree with S19 (−2), indicating that while they saw the need to share personal contact information to facilitate communication with parents and students, they felt burnout from rude behaviour such as contacting them after hours or on weekends.

Type 4 teachers experience burnout due to student indifference and the excessive emphasis on student rights. Disrespectful student behaviour and lack of class participation diminish the teacher’s passion for education, contributing significantly to teacher burnout. This attitude creates a sense of helplessness regarding their work. Type 4 teachers also feel that the excessive emphasis on student rights undermines their authority during the guidance process. They struggle with a loss of self-esteem when they cannot appropriately address student behavioural issues. South Korea’s cultural context amplifies these challenges, as the increasing focus on student rights often clashes with traditional expectations of teacher authority and respect.

## 5. Discussion

This study explored the types of burnout experienced by teachers and the characteristics of each type. The findings identified four distinct teacher burnout types, each influenced by unique personal, organisational and cultural factors.

Teachers in the type ‘burnout due to damaged self-esteem’ (Type 1) experience burnout stemming from feelings of humiliation and a loss of professional identity, often caused by rude behaviours from students and parents. This finding aligns with research indicating that damaged self-esteem contributes to reduced job satisfaction and increased turnover rates [56].

In Korea, the cultural emphasis on hierarchical respect further complicates this issue. When students and parents treat teachers as service providers rather than professionals, it exacerbates feelings of inadequacy. Successful programmes, such as the ‘Mindfulness-Based Stress Reduction (MBSR)’ piloted in Gyeonggi-do, have shown positive outcomes in helping teachers rebuild self-esteem. Expanding this model nationwide could provide targeted support for Type 1 teachers. Similarly, the ‘Teachers’ Healing Centre’ initiative in Seoul offers regular counselling services. Enhancing the programme by adding peer support groups and one-on-one coaching sessions could further strengthen community resilience [57,58]. In addition, individual interventions for reducing teacher burnout, such as programmes for developing emotional intelligence, enhancing resilience and improving stress management skills, can effectively alleviate teacher burnout [59]. For the successful implementation of these programmes, it is essential to train expert counsellors specialising in schools and the teaching profession.

Teachers experiencing ‘burnout due to organisational neglect’ (Type 2) feel burnout when others undermine their authority, and they receive little institutional support. This issue arises from a broader organisational failure to include teachers in decision-making and policy development, leaving them feeling helpless and frustrated [60].

In Korea, while the Teacher Status Act aims to protect teachers, it often lacks effective enforcement mechanisms. For example, during cases of school violence or parental complaints, many teachers report inadequate support from administrators [61]. The government could revise the Act to include explicit punitive measures for violations of teacher authority and better support mechanisms during school incidents. Successful examples from other contexts, such as the U.S. Teacher Protection Act, which allows teachers to discipline students without legal liability, could inform these reforms [62].

Active interactions within and outside the system are necessary to improve the quality of education [63]. Research indicates that inadequate institutional support contributes significantly to teacher burnout [25], whereas educational institutions employing teachers with high levels of well-being tend to cultivate more collaborative and positive organisational environments [64]. In addition, empowering teacher unions to participate in policy discussions and creating platforms for regular consultations between teachers and administrators could address organisational neglect. Additionally, formal recognition programmes for innovative teaching practices and annual awards could enhance motivation and collaboration among teachers [65].

Teachers experiencing ‘burnout due to excessive role demands’ (Type 3) face burnout due to unrealistic societal expectations and role overload, including additional administrative tasks and responsibilities for students’ character development, which often go beyond their formal duties. One effective approach to mitigating teacher burnout is enhancing work–life balance. By implementing strategic task management and offering flexible scheduling, schools can significantly reduce educator stress and boost overall job satisfaction [59]. However, teachers in Korea are vulnerable due to the dual burden of administrative work and classroom management. For instance, homeroom teachers must often resolve parental complaints, which is a task more appropriate for senior administrators [31]. This situation highlights a need for clear role definitions and equitable workload distribution within schools.

Programmes such as parental education workshops piloted in Seoul have successfully recalibrated parents’ expectations and encouraged shared accountability in education. Expanding these workshops nationwide could foster mutual respect between teachers and parents. Additionally, mentoring programmes for early-career teachers and ongoing professional development initiatives are essential for building resilience and reducing burnout [66].

Finally, teachers experiencing ‘burnout due to disrespectful behaviour’ (Type 4) do so when students show indifference or engage in disrespectful behaviours. This finding aligns with studies indicating that rude student behaviour significantly increases emotional burnout and depersonalisation among teachers [67,68].

In Singapore, teachers cultivate tailored educational approaches to address the diverse backgrounds and needs of their students by undergoing training in cultural sensitivity and inclusive teaching practices [69]. In addition, programmes like ‘Student–Teacher Dialogue Days’, introduced in Daejeon, have shown promise in bridging the communication gap between students and teachers. These initiatives foster mutual understanding and respect, reducing burnout caused by classroom disruptions. Regular one-on-one meetings and project-based learning could further enhance student engagement and minimise indifference.

Teachers also need training in emotional regulation and behaviour management techniques to address student indifference. Building trust between teachers and students is crucial, as it fosters a positive classroom atmosphere and reduces teacher burnout.

## 6. Conclusions

This study’s results highlight how cultural and institutional factors influence teacher burnout. For example, teachers in the ‘burnout due to self-esteem type’ (Type 1) reflect the Korean cultural emphasis on respect and professional identity, which clashes with modern challenges in teacher–parent dynamics. Teachers experiencing ‘burnout due to organisational neglect’ (Type 2) illustrate how hierarchical organisational structures in Korean schools exacerbate feelings of helplessness. Similarly, those experiencing ‘burnout due to excessive role demands’ (Type 3) highlight societal pressures for teachers to perform roles beyond their expertise, while teachers experiencing ‘burnout due to disrespectful behaviour’ (Type 4) underscore the growing emphasis on student rights, which sometimes undermines teacher authority.

Addressing burnout requires tailored solutions that consider these cultural contexts. For example, revising teacher training programmes to include strategies for navigating cultural challenges, enhancing teacher autonomy and promoting mutual respect between stakeholders could mitigate burnout more effectively.

Based on this study’s results, we recommend the following strategies to prevent and mitigate teacher burnout.

First, strengthen interpersonal relationships: Develop evidence-based programmes to enhance teacher–student engagement and build trust among colleagues. Peer mentoring, regular communication sessions and conflict resolution training could improve workplace relationships.

Second, recalibrate expectations: Implement parental education programmes to adjust unrealistic expectations. Workshops and public campaigns should emphasise shared accountability in education.

Third, enhance institutional support: Revise the Teacher Status Act to include explicit measures for protecting teacher authority. Establishing a dedicated hotline for reporting violations and offering immediate legal support could strengthen institutional protections.

Fourth, promote sociocultural change: Launch campaigns that promote respect for teachers at home and in schools. Public recognition of teachers’ contributions and stricter penalties for verbal or physical abuse could enhance their professional standing.

Finally, addressing teacher burnout requires a comprehensive approach that integrates cultural awareness, institutional reforms and proactive engagement with all stakeholders. By implementing this study’s recommendations, schools and policymakers can create a more supportive environment that fosters teacher well-being and enhances the quality of education.

## 7. Limitations

This study used the Q methodology to categorise the various types of burnout experienced by teachers and explore their characteristics. However, there are several limitations:

First, while the sample size of 30 participants is acceptable for a Q methodology, it is relatively small and may not adequately represent the broader population of Korean teachers. This limitation raises concerns about the statistical generalisability of the findings. Another potential limitation is the small sample size and the potential influence of cultural biases on participants’ responses. Therefore, future research should include quantitative studies examining factors such as teachers’ gender, experience, work environment and school characteristics that influence each type. Additionally, researchers might consider conducting longitudinal studies or cross-cultural comparisons to provide more robust insights and enhance generalisability.

Second, the Q methodology involves participants expressing subjective opinions on presented statements, which may not fully capture the complex aspects of burnout experienced by teachers. In particular, qualitative research methods such as narratives could complement the in-depth exploration of burnout’s emotional and psychological aspects.

Third, while this study provides valuable insights into teacher burnout, its cross-sectional design does not account for changes in burnout patterns over time. Teacher burnout can evolve across different career stages and in response to shifts in educational policies or social environments, potentially influencing its degree and types. Future research should consider adopting longitudinal or experimental designs to capture burnout’s dynamic nature, analyse its underlying causes and evaluate the effectiveness of interventions.

Finally, this study lacks comparative insights across diverse cultural or educational contexts. Teacher burnout may manifest differently based on international differences in policies, societal attitudes and school environments. Thus, comparative research across various countries could provide a more comprehensive understanding and enable the development of globally relevant solutions.

By addressing these limitations, future studies can enrich the theoretical and practical contributions in order to mitigate teacher burnout effectively.

## Figures and Tables

**Table 1:** Demographic background and factor weights by type.

Type	No.	Weight	School Level	Gender	School Type	Teaching Years	Region	Subject	Age
Type 1 (N = 9)	P11	10	High school	Female	Private	21	Gyeonggi	Career	47
	P14	10	Middle school	Female	Public	15	Gyeonggi	Society	46
	P4	2.85764	Middle school	Female	Public	20	Gyeonggi	Math	48
	P22	2.28672	High school	Male	Private	11	Seoul	Physics	41
	P18	2.25649	Middle school	Male	public	1	Gyeonggi	Home Economics	27
	P1	2.11011	High school	Female	Public	15	Gyeonggi	Chinese	23
	P15	1.65303	High school	Female	Private	20	Seoul	Science	51
	P27	1.20072	Middle school	Female	Public	2.6	Gyeonggi	Music	30
	P16	0.61227	High school	Male	Public	2	Gyeonggi	Biology	29
Type 2 (N = 8)	P26	4.33816	High school	Male	Private	18	Seoul	Society	46
	P7	3.84102	High school	Male	Public	8	Gyeonggi	Math	36
	P12	3.21178	High school	Female	Private	21	Seoul	Math	51
	P13	2.86707	High school	Female	Private	19	Seoul	Ethics	52
	P3	2.4256	High school	Female	Private	7	Seoul	Physics	36
	P20	1.64409	Middle school	Female	Public	6.5	Gyeonggi	Home Economics	29
	P30	1.13859	Middle school	Female	Public	20	Seoul	Math	58
	P29	1.10706	Middle school	Female	Public	1.8	Gyeonggi	Math	30
Type 3 (N = 6)	P28	4.51538	Middle school	Female	Public	10	Seoul	Home Economics	42
	P17	3.35335	Middle school	Male	Private	17	Seoul	Technology	44
	P25	2.33583	High school	Male	Private	12	Seoul	Math	40
	P2	2.03878	Middle school	Male	Private	9	Seoul	Math	37
	P19	1.85507	Middle school	Male	Public	0.5	Gyeonggi	Physical Education	27
	P10	1.57046	Middle school	Female	Public	4	Gyeonggi	Home Economics	30
Type 4 (N = 7)	P8	2.04755	Middle school	Female	Public	24	Gyeonggi	Korean Language	50
	P23	1.85312	Middle school	Male	Private	18	Seoul	English	49
	P6	1.71067	High school	Female	Public	12	Gyeonggi	English	44
	P9	1.64245	Middle school	Male	Private	6	Seoul	Technology	34
	P24	1.59924	Middle school	Male	Private	14	Seoul	Society	43
	P5	1.5407	Middle school	Female	Public	11	Gyeonggi	Science	41
	P21	1.48056	High school	Female	Public	9	Gyeonggi	Art	37

**Table 2:** Eigenvalues and explained variances for four factors.

	Factor 1	Factor 2	Factor 3	Factor 4
Eigenvalue	7.373851	3.767569	2.6625	2.0241
% Explained Variance	25	13	9	7
Cumulative % Explained Variance	25	38	47	54

**Table 3:** Z-scores and Q-sort values of the statements for each factor.

No.	Statement	Factor 1		Factor 2		Factor 3		Factor 4	
		Z-Score	Q-Sort Value	Z-Score	Q-Sort Value	Z-Score	Q-Sort Value	Z-Score	Q-Sort Value
S1	I feel frustrated with a teaching organisation that does not prioritise the well-being of teachers.	−0.14	0	1.37	3 *	0.2	0	0.71	1
S2	There is a need for legal measures to limit parents’ excessive expectations and demands.	1.19	2	0.01	0 *	0.77	2	0.98	2
S3	I feel humiliated by the rude attitudes of students and parents.	1.24	3	1.2	3	1.43	3	−0.07	0 *
S4	I am exhausted by the feeling that people see me only as someone with teaching duties.	−1.65	−4 *	0.17	0	−0.08	0	−0.48	−1
S5	I feel insignificant when I think about the level of welfare for teachers.	−1.16	−2	−1.12	−2	0.44	1 *	−0.94	−2
S6	The excessive emphasis on student rights compared to teacher authority is problematic.	−1.53	−3 *	0.49	1	0.88	2	1.84	4 *
S7	I feel frustrated when student misconduct is blamed on teachers.	2.04	4	1.16	2	1.66	4	1.02	3
S8	Teachers should not have to perform school duties outside of working hours.	−0.01	0 *	0.87	2	1	2	−0.99	−2 *
S9	I get angry when fellow teachers disregard my subject.	−1.83	−4	0.86	2 *	−0.38	−1 *	−1.33	−3
S10	Family support greatly enhances a teacher’s resilience against psychological burnout.	−1.37	−3	0.8	1 *	−0.08	0 *	−1.06	−2
S11	There’s not much I can do for fellow teachers who suffer from infringement of their authority in the school field.	0.09	0	1.49	4 *	0.6	1	0.42	1
S12	The administrator’s passive response to complaints lowers motivation.	1.42	3	1.93	4	1.22	3	0.74	1
S13	I experience psychological burnout when I lose confidence in my work.	−0.16	−1	−0.76	−2	−1.79	−4	−1.79	−4
S14	I feel anxious about how long I can continue my teaching career.	−0.08	0	−1.41	−3 *	−0.37	−1	−0.29	−1
S15	To prevent teacher burnout, I cannot correct every mistake students make.	−0.07	0	−1.21	−3 *	−0.38	−1	−0.21	−1
S16	It’s wrong to make teachers responsible for students’ character development.	0.28	1 *	−0.8	−2	1.13	3 *	−0.4	−1
S17	There is a need to define the scope of teachers’ duties.	1.08	2	0.65	1	−0.29	−1	0.15	0
S18	I feel burned out when others demand only exemplary behaviour from teachers.	−1.41	−3	−0.38	−1	−0.47	−2	−1.76	−4
S19	Schools should not offer teachers’ personal contact information to students or parents.	0.37	1	0.43	0	−0.12	0	−1.04	−2 *
S20	Psychological burnout occurs due to conflicts among colleagues.	−0.42	−1	−0.37	0	−0.64	−2	−0.85	−1
S21	Social distrust towards teachers causes psychological burnout.	−0.72	−2	−0.28	0	−0.1	0	0.59	1
S22	Most current measures to prevent teachers’ psychological burnout are ineffective.	0.49	1	0.93	2	−0.02	0	1.05	3
S23	Unreasonable demands to include specific content in student records diminish my motivation.	0.22	0	−0.27	0	−1.61	−3 *	0.1	0
S24	Excessive interest in my private life makes me tired.	−0.96	−2	−0.67	−1	−1.03	−2	0.18	0 *
S25	Receiving indiscriminate KakaoTalk messages or texts annoys me.	0.25	1	−0.79	−2 *	0.56	1	0.89	2
S26	I experience psychological burnout when I see students doing academy homework during class.	0.52	1	−0.44	−1 *	0.54	1	1.25	3 *
S27	I experience psychological burnout when I see students openly sleeping during class.	0.98	2 *	−0.39	−1	−0.95	−2	1.7	4 *
S28	Meaningless administrative tasks exhaust me.	0.53	2	−0.74	−1 *	0.99	2	0.8	2
S29	It is unfair for administrators to assign tasks unilaterally without considering my aptitude or circumstances.	−1.02	−2	1.18	3 *	−1.38	−3	0.01	0 *
S30	Distrust in public education causes psychological burnout for teachers.	−0.34	−1	−1.69	−4 *	−0.39	−1	0.77	1 *
S31	If teachers’ salaries and treatment improve, teacher authority will increase.	−0.33	−1 *	−1.43	−3	1.75	4 *	−1.29	−3
S32	I feel frustrated when I think I lack competence as a teacher.	1.24	3 *	0.53	1	−1.94	−4 *	0	0
S33	I feel insignificant when I compare myself to other teachers who seem superior.	−0.31	−1 *	−1.84	−4	−1.68	−3	−1.61	−3
S34	I feel ashamed when parents or students view the school as a service institution.	1.57	4 *	0.49	1	0.52	1	0.89	2

* *p* < 0.05.

## Data Availability

The data are available from the corresponding author upon reasonable request.
